# Anti-HIV reverse transcriptase plant polyphenolic natural products with in silico inhibitory properties on seven non-structural proteins vital in SARS-CoV-2 pathogenesis

**DOI:** 10.1186/s43141-021-00206-2

**Published:** 2021-07-16

**Authors:** Von Novi O. de Leon, Joe Anthony H. Manzano, Delfin Yñigo H. Pilapil, Rey Arturo T. Fernandez, James Kyle Anthony R. Ching, Mark Tristan J. Quimque, Jay Carl M. Agbay, Kin Israel R. Notarte, Allan Patrick G. Macabeo

**Affiliations:** 1 Laboratory for Organic Reactivity, Discovery and Synthesis (LORDS), Research Center for the Natural and Applied Sciences, University of Santo Tomas, España Blvd., 1015 Manila, Philippines; 2 Department of Biological Sciences, College of Science, University of Santo Tomas, España Blvd., 1015 Manila, Philippines; 3 Department of Chemistry, College of Science, University of Santo Tomas, España Blvd., 1015 Manila, Philippines; 4 The Graduate School, University of Santo Tomas, España Blvd., 1015 Manila, Philippines; 5 Chemistry Department, College of Science and Mathematics, Mindanao State University – Iligan Institute of Technology, Tibanga, 9200 Iligan City, Philippines; 6 Philippine Science High School – Central Mindanao Campus, 9217 Balo-I, Lanao del Norte, Philippines; 7 Faculty of Medicine and Surgery, University of Santo Tomas, España Blvd., 1015 Manila, Philippines

**Keywords:** SARS-CoV-2, Non-structural proteins, Molecular docking, ADMET, Polyphenolics, Terpenoids, Alkaloid, HIV reverse transcriptase

## Abstract

**Background:**

Accessing COVID-19 vaccines is a challenge despite successful clinical trials. This burdens the COVID-19 treatment gap, thereby requiring accelerated discovery of anti-SARS-CoV-2 agents. This study explored the potential of anti-HIV reverse transcriptase (RT) phytochemicals as inhibitors of SARS-CoV-2 non-structural proteins (nsps) by targeting in silico key sites in the structures of SARS-CoV-2 nsps. One hundred four anti-HIV phytochemicals were subjected to molecular docking with nsp3, 5, 10, 12, 13, 15, and 16. Top compounds in complex with the nsps were investigated further through molecular dynamics. The drug-likeness and ADME (absorption, distribution, metabolism, and excretion) properties of the top compounds were also predicted using SwissADME. Their toxicity was likewise determined using OSIRIS Property Explorer.

**Results:**

Among the top-scoring compounds, the polyphenolic functionalized natural products comprised of biflavones **1**, **4**, **11, 13**, **14**, **15**; ellagitannin **9**; and bisisoquinoline alkaloid **19** were multi-targeting and exhibited strongest binding affinities to at least two nsps (binding energy = − 7.7 to − 10.8 kcal/mol). The top ligands were stable in complex with their target nsps as determined by molecular dynamics. Several top-binding compounds were computationally druggable, showed good gastrointestinal absorptive property, and were also predicted to be non-toxic.

**Conclusions:**

Twenty anti-HIV RT phytochemicals showed multi-targeting inhibitory potential against SARS-CoV-2 non-structural proteins 3, 5, 10, 12, 13, 15, and 16. Our results highlight the importance of polyhydroxylated aromatic substructures for effective attachment in the binding/catalytic sites of nsps involved in post-translational mechanism pathways. As such with the nsps playing vital roles in viral pathogenesis, our findings provide inspiration for the design and discovery of novel anti-COVID-19 drug prototypes.

**Supplementary Information:**

The online version contains supplementary material available at 10.1186/s43141-021-00206-2.

## Background

The rapid spread of the severe acute respiratory syndrome coronavirus 2 (SARS-CoV-2) marks itself as one of the deadliest viruses in recent history due to its high mortality and morbidity rates [1, 2]. As of May 2021, the World Health Organization recorded over one hundred sixty-seven million cases worldwide with 3.4 million deaths [3]. Continuous efforts are being carried out to unravel the pathophysiology of the virus, paving the way to the discovery and development of efficacious vaccines and anti-SARS-CoV-2 drugs. While the world continues to make strides in vaccine development and rollout, drug-based treatments are still needed to cure the growing number of COVID-19-afflicted individuals. Thus, developing effective therapeutic agents against SARS-CoV-2 remains a global health need.

The discovery of antiviral chemotherapeutic prototypes requires accurate identification of drug targets. Among which, the SARS-CoV-2 non-structural proteins (nsps) are among the highly favored targets because of their role in viral replication, post-translational mechanisms, and host immunity evasion that influence SARS-CoV-2 virulence and pathogenesis [4]. The repurposing of bioactive natural products is one of the key strategies available for screening potential SARS-CoV-2 nsps inhibitors. To date, plant-based medicines as treatment for SARS-CoV-2 infection have not been reported.

**Fig. 1:**
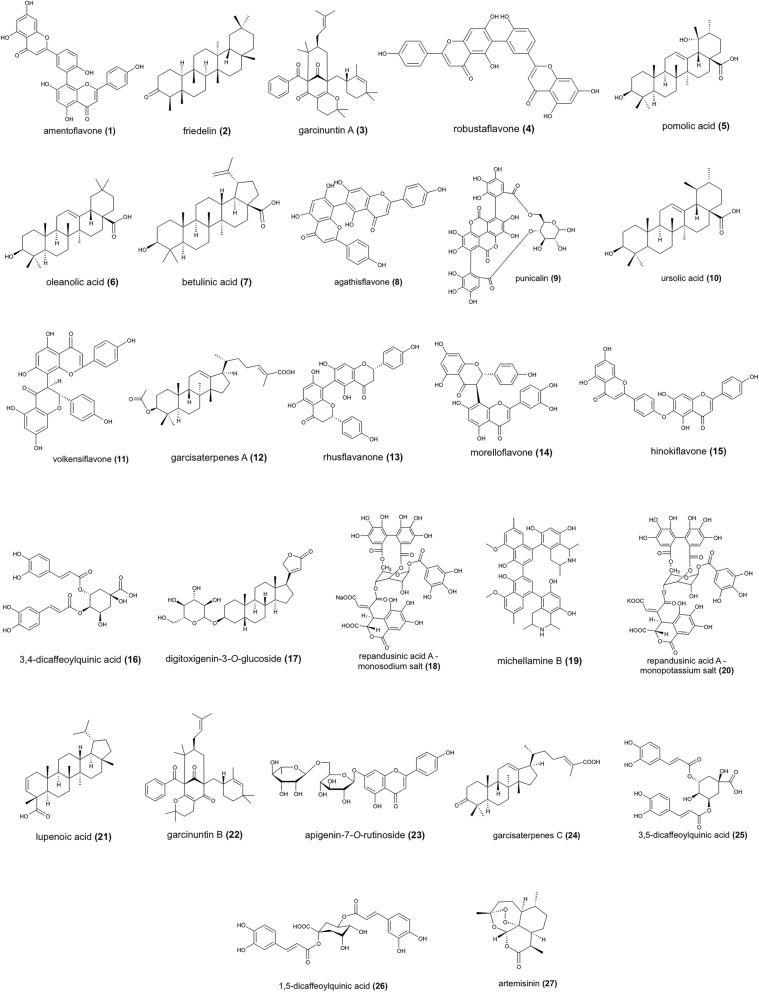
Anti-HIV RT phytochemicals with strong binding affinities to at least one of the target nsps

Plant-derived natural products are established biotechnological-derived substances that exhibit a wide range of biological activity including antagonistic properties against human immunodeficiency virus (HIV) and coronaviruses such as Middle East respiratory syndrome coronavirus (MERS-CoV) and SARS-CoV [5–9]. Relevant to this study, polyphenolic natural products such as flavonoids and tannins (Fig. 1) are well-recognized to confer broad-spectrum antiviral activities in addition to possessing anti-inflammatory, anti-tumor, antioxidants, immune, and prebiotic properties [10]. Recent studies in anti-COVID-19 drug discovery have highlighted the potential of polyphenolic compounds through in silico-guided investigations against protein targets in SARS-CoV-2 involved in infective mechanisms, i.e., inhibition of spike (*S*) protein, angiotensin-converting enzyme 2 (ACE-2) receptor, papain-like protease (PLpro), 3-chymotrypsin-like cysteine protease (3CLpro), and RNA-dependent-RNA-polymerase (RdRp) [10–13]. In addition, computational studies describing the potential of other classes of natural products as SARS-CoV-2 3CLpro, PLpro, and RdRp protein inhibitors have been reported [5, 14–19].

Considering the similarity between SARS-CoV-2 and HIV, we repurposed previously reported anti-HIV reverse transcriptase (RT) secondary compounds using in silico simulations in this study. SARS-CoV-2 and HIV are single-stranded RNA viruses that utilize RNA-dependent polymerases and code precursor polyproteins vital for their respective pathogenesis. In this paper, we disclose computational interrogation of 104 known anti-HIV RT phytochemicals against seven target proteins, namely nsp3 (PLpro), nsp5 (3CLpro), nsp12 (RdRp), nsp13 (helicase), nsp15 (endoribonuclease), and the nsp16-nsp10 complex (*S*-adenosylmethionine complex). The thermodynamic stability and the pharmacokinetic characteristics of the top-ranked compounds are also reported.

## Methods

### Target enzyme preparation

Seven target enzymes with important functions in SARS-CoV-2 infectivity were selected and obtained from the Protein Data Bank (PDB): 3CLpro (PDB ID: 6LU7), PLpro (PDB ID: 6W9C), RdRp (PDB ID: 6M71), helicase (6JYT), nsp16-nsp10 complex (6W4H), and nsp15 (6VWW). These proteins in three-dimensional structures were added to UCSF Chimera 1.14 platform as PDB files [20]. All proteins belong to SARS-CoV-2 except for helicase due to unavailability of nsp13. Thus, helicase model from SARS-CoV-1 which shares 99.8% sequence identity and 100% sequence similarity with that of SARS-CoV-2 was used [21]. Coronavirus helicase domains are distinct compared to other (+)-sense RNA virus domains due to the presence of linkage in a single protein to a binuclear zinc-binding domain at the N-terminus. This domain is composed of 12 conserved cysteine-histidine residues and is a good target in antiviral drug discovery [22–25].

### Ligand selection and preparation

A total of 104 plant secondary metabolites (Supplementary Figure 1; Supplementary Table 1) previously reported to inhibit HIV RT [25] were used as ligands targeting the above-mentioned viral proteins. The plant metabolite structures were formatted as SYBYL mol2 file or in SMILES notation using Avogadro (version 1.20) and were added to the UCSF Chimera 1.14 platform [26].

### Molecular docking simulations

Molecular docking experiments were carried out on UCSF Chimera 1.14 platform with AutoDock Vina plugged-in as docking algorithm [20]. Protein structures in three dimensions were opened in PDB formats. Co-crystallized ligands and other molecules were removed from the crystallized protein. Ligands were added in the platform as SYBYL mol2 files or in SMILES notation. Ligand and protein structures were minimized through addition of missing hydrogen atoms and charges to the structures using the Gasteiger charge method, which was computed using Amber’s Antechamber module [27]. ‘Flexible ligand into flexible active site’ protocol was followed during execution of docking procedures. In this protocol, flexible ligands were allowed and positioned within a grid box (Supplementary Table 2) which encompasses the enzymatic ligand-binding cavity, as predicted using COACH algorithms [28].

### Druggability, ADME, and toxicity prediction

Top ten compounds per nsp, which in total were twenty-seven compounds, were selected for druggability, pharmacokinetic, and toxicity analyses. Absorption, distribution, metabolism, and excretion (ADME) properties of top twenty-seven compounds overall were computationally predicted using SwissADME software. Evaluation of pharmacokinetic profiles of compounds was performed according to Lipinski's ‘rule of five’ which assesses biochemical properties of a drug candidate involved in permeation and cell absorption. Three of the following values need to be met according to Lipinski’s criteria: < 500 Daltons (Da) for molecular weight, < 5 for calculated lipophilicity (MLogP), < 10 for the number of hydrogen-bond acceptors, and < 5 for the number of hydrogen bond donors [29]. Moreover, toxicity of hit compounds, specifically mutagenicity, tumorigenicity, reproductive toxicity, and irritant effects, were predicted in silico using OSIRIS Property Explorer software [30]. Solubility (LogS) was also predicted using the same software in which LogS ≥ − 4 indicates good solubility and favorable absorption of compounds.

### Molecular dynamics simulations

Molecular dynamics (MD) simulation was employed to understand the dynamic behavior of the top-binding complexes based on molecular docking analysis. All MD simulations were carried out using GROMACS version 2020.1 under the Ubuntu Linux platform version 2020.1-1 [31]. The SARS-CoV-2 non-structural protein topologies were generated using the CHARMM 36 force field with TIP3P water model, while ligand topology was generated using CGenFF (CHARMM general force field). The complex was solvated on a dodecahedron grid by single point charge (SPC) water. The system was then neutralized with counterions. Energy minimization was done on the system using the steepest descent integrator for 5000 steps and Particle Mesh Ewald (PME) algorithm for the Coulomb and van der Waals interactions [32]. After system equilibration, each system was subjected to molecular dynamic simulation for 20 ns at constant temperature of 300 K. The dynamic trajectories were recorded during the production every 0.01 ns which were used to analyze the root mean square deviation (RMSD) and root mean square fluctuation (RMSF) for each system.

## Results

One hundred four repurposed anti-HIV reverse transcriptase phytochemicals against SARS-CoV-2 nsps comprised of polyphenolics, terpenoids, alcohols, and alkaloids were docked with nsps3, 5, 10, 12, 13, 15, and 16. Twenty-seven compounds, which are included in the top 10 compounds per nsp, showed favorable binding affinities (Fig. 1). In addition, twenty of the top compounds exhibited multi-targeting properties.

### Molecular docking with autolytic-processing enzymes (nsp3 and nsp5)

**Fig. 2:**
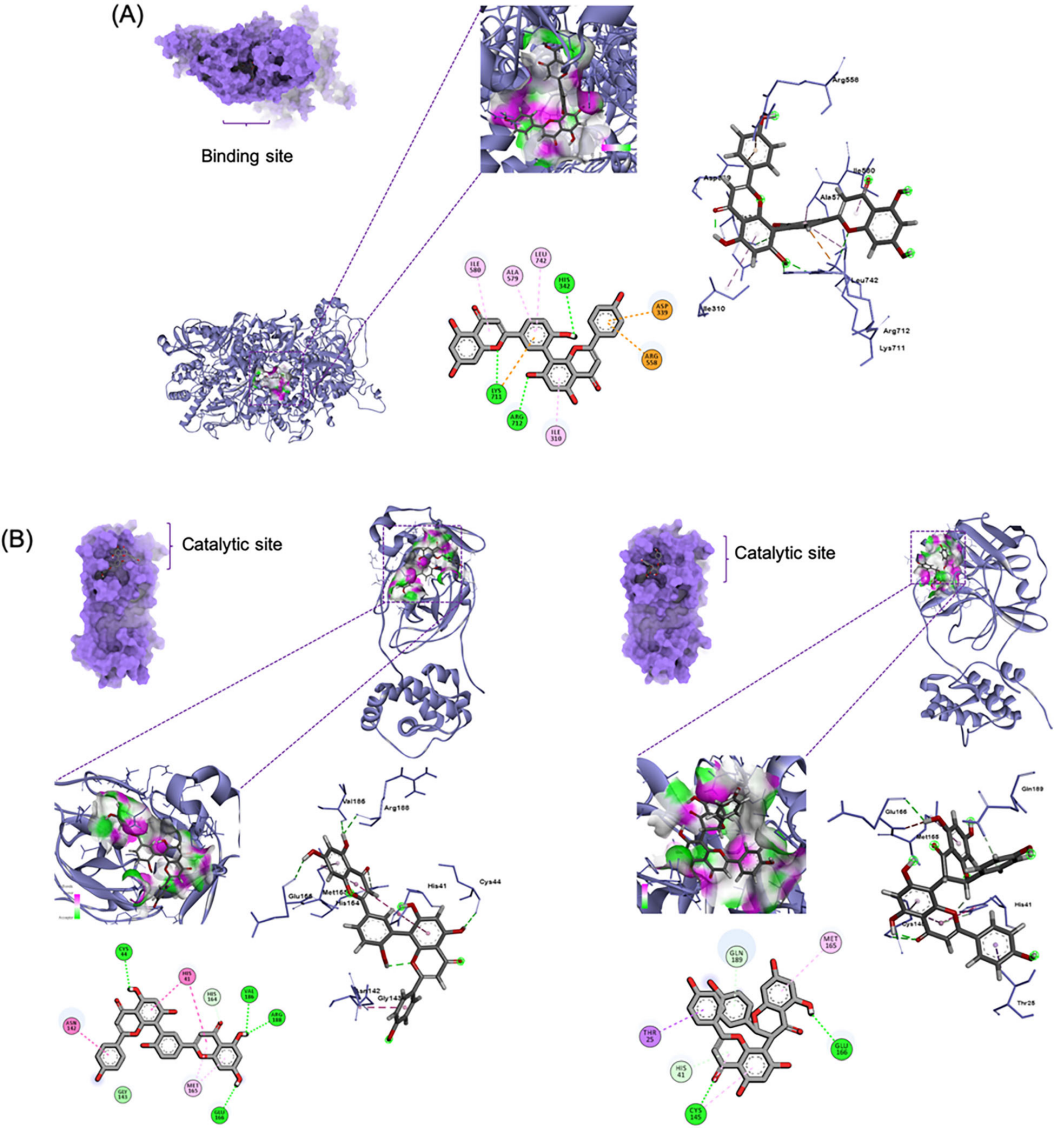
Top 1-binding compounds in complex with their target cysteine proteases: **A** amentoflavone (**1**) in complex with PLpro, **B** amentoflavone (**1**) (left) and volkensiflavone (**11**) (right) in complex with 3CLpro

Top ten compounds against PLpro exhibited binding affinities of − 10.1 to − 10.8 kcal/mol (Table 1). The biflavonoid amentoflavone (**1**) exhibited highest affinity to PLpro with its benzopyrone (ring C) and phenolic moiety (ring B) participating through H-bonding (5.62 Å) and *pi*-anion interactions with Lys711, respectively (Fig. 2A). Ring C additionally bound Ile580 through *pi*-alkyl interaction. The phenolic functionality in ring B also participated in hydrogen bonding with His342 (3.49 Å) and in *pi*-alkyl binding with Ala579 and Leu742. Ring A’ of the benzopyrone moiety bound Arg712 by H-bonding (5.72 Å) and Ile310 by *pi*-alkyl interaction. Meanwhile, the phenolic moiety (ring B’) exhibited *pi*-anion interaction with Asp339 and *pi*-cation interaction with Arg558.

**Table 1:** Binding affinities and interactions of top ten ligands against the cysteine proteases

Target	Cpd	Binding affinity (kcal/mol)	Hydrogen bonds	Other interactions
PLpro	**1**	− 10.8	His342, Lys711, Arg712	Lys711, Asp339, Arg558, Ile310, Ile580, Ala579, Leu742
	**2**	− 10.7	None	His342, Leu557, Ala579, Leu742
	**3**	− 10.7	Lys711, Arg712	Ile310, Ala338, His342, Leu557, Ala579, Ile580, Val635, Lys694, Arg712
	**4**	− 10.6	Thr583, Arg586, Tyr634	Asp339, Arg558, Ala579, Ile580, Met630, Leu742
	**5**	− 10.4	Val659	Leu557, Arg558, Met560, Ala579, Ile580, Leu742
	**6**	− 10.3	Asp226	None
	**7**	− 10.2	Lys711, Arg712	None
	**8**	− 10.2	Asp339, Arg586, Tyr634	Val304, Ala338, Asp339, Arg558, Ala579, Lys711, Leu742
	**9**	− 10.2	Gly337, Asp339, Arg345, Arg558, Arg712	Ile310
	**10**	− 10.1	Asp339, Arg345, Tyr634	Leu557, Ile580, Met630, Val635, Lys711, Leu741
3CLpro	**1**	− 8.6	Cys44, Val186, Arg188, Glu166	Thr25, His41, Asn142, Cys145, Met165
	**11**	− 8.6	Cys145, Glu166	Thr25, His41
	**12**	− 8.5	Thr24, Ser46, Thr190, Gln192	Thr25, His41
	**13**	− 8.5	Thr26, His41	Met49, Pro168
	**4**	− 8.5	Arg188, Gln189	None
	**8**	− 8.4	Thr26, Gln189, Thr190	Leu27, Met49, Glu166, Met165, Pro168
	**14**	− 8.4	Phe140, Gly143, Arg188, Gln189	His41
	**15**	− 8.1	Asn119, Val186	None
	**16**	− 7.9	Gly143, Cys145, Glu166, Gln189	His41, Gln189
	**17**	− 7.9	His41, Asn119	His41, Gly143, His163
	**3**	− 7.9	His41	Leu27, His41

On the other hand, top-ranked ligands against 3CLpro exhibited binding affinities of − 7.9 to − 8.6 kcal/mol (Table 1). The biflavones amentoflavone (**1**) and volkensiflavone (**11**) showed highest affinity to 3CLpro (Fig. 2B). The chromanone moieties (rings A′ and C) of amentoflavone showed stacked amide-*pi* and *pi*-*pi* T-shaped interactions with His41, a component of the 3CLpro catalytic dyad. These interactions were also demonstrated by its phenolic moiety to Asn142. The chromanone moiety (rings A and C) was bound to Met165 through *pi*-alkyl interaction along with hydrogen bonds with Val186 (5.46 Å), Arg188 (5.86 Å), and Glu166 (4.83 Å). Ring A′ also bound Cys44 through H-bonding (4.77 Å). Volkensiflavone (**11**) was likewise bound to the 3CLpro catalytic dyad, His41 and Cys145, through *pi*-anion interaction and hydrogen bonding (3.71 Å) of the chromanone moieties (rings C and A′ respectively). Moreover, rings A′ of the flavone substructure also exhibited hydrogen bonding with Glu166 (3.88 Å) while the B ring residue bound Thr25 through a *pi*-sigma interaction.

### Molecular docking with replication-transcription complex enzymes (nsp12 and nsp13)

**Fig. 3:**
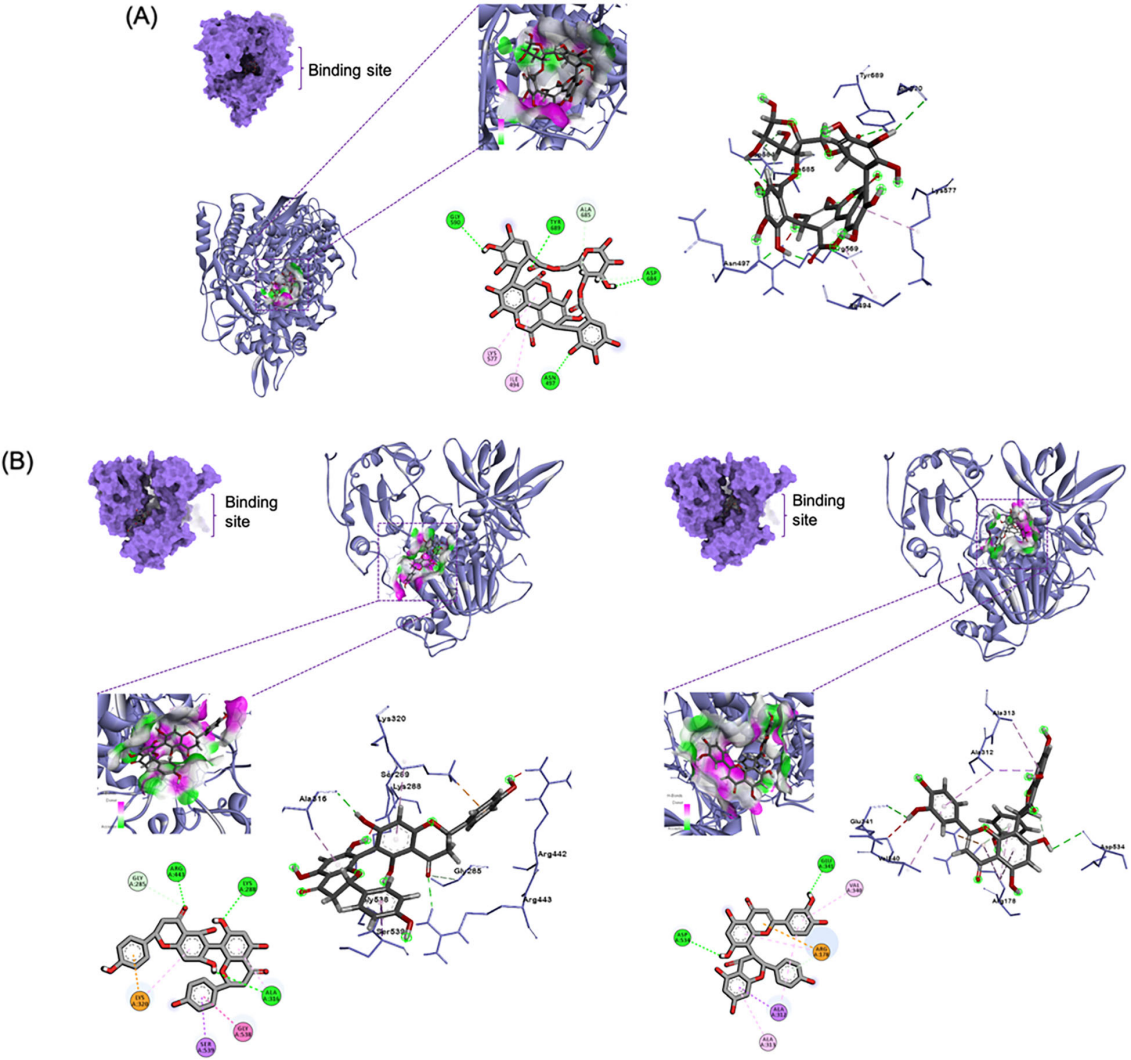
Top 1-binding compounds in complex with their target enzymes vital in replication: **A** punicalin (**9**) in complex with RdRp, **B** rhusflavanone (**13**) (left) and morelloflavone (**14**) (right) bound to helicase

Among the top ten compounds against RdRp with binding affinities of − 8.6 to − 9.5 kcal/mol, the ellagitannin punicalin (**9**) exhibited the highest affinity (Table 2; Fig. 3A). The ellagic acid moiety occupied Ile494 and its galloyl hydroxyl bound Asn497 (4.13 Å), which are both components of the RdRp finger domain that is responsible for the entry and exit of the RNA template during replication-transcription [33]. Moreover, its glucose hydroxyl and hydrogen participated in hydrogen bonding (4.79 Å) and carbon-hydrogen bonding respectively with Asp684, a component of the motif B of the polymerase active site [34]. Other interactions include the participation of its ellagic acid moiety in *pi*-alkyl interaction with Lys577, galloyl hydroxyl in hydrogen bonding with Gly590 (2.87 Å), carbonyl oxygen in hydrogen bonding with Tyr689 (5.76 Å), and glucose moiety in carbon-hydrogen bonding with Ala685.

**Table 2:** Binding affinities and interactions of top ten ligands against the nsps vital for replication

Target	Cpd	Binding affinity (kcal/mol)	Hydrogen bonds	Other interactions
RdRp	**9**	− 9.5	Asn497, Gly590, Asp684, Tyr689	Ile494, Lys577, Asp684, Ala685
	**10**	− 9.1	Val495	Ile494, Lys577, Ala580, Ala685
	**2**	− 8.9	None	Ile494, Arg569, Leu576, Lys577, Ala685
	**15**	− 8.9	Asn496, Asn497, Arg569, Ala685	Ile494, Lys500, Lys577, Ala580, Ala685
	**18**	− 8.9	Asn496, Arg569, Ala685, Ser759	Lys545, Arg569
	**19**	− 8.8	Ile548, Lys593, Ser814	Ile548, Lys593, Leu758, Asp761, Cys813, Pro832, Arg836, Ile837, Ala840
	**20**	− 8.8	Ile494, Asp684	Lys500, Lys545, Arg569, Ser682
	**4**	− 8.8	Asn497, Arg569	Ile494, Lys500, Arg569, Lys577, Ala685
	**21**	− 8.7	None	Ile494, Lys500, Leu576, Lys577, Ala685, Tyr689
	**1**	− 8.6	Asn497, Asp684	Arg569, Ala580, Ala688, Tyr689
	**3**	− 8.6	Arg569, Gln573	Ile494, Lys500, Lys577, Ala580, Ile589, Ala685, Ala688, Tyr689
	**5**	− 8.6	Arg569, Gln573	Leu576, Lys577, Ala580, Ala685
Helicase	**14**	− 9.2	Glu341, Asp534	Ala312, Ala313, Val340
	**13**	− 9.2	Lys288, Ala316, Arg443	Thr286, Ala316, Lys320, Gly538, Ser539
	**15**	− 9	Arg332, Glu319, Cys342, Ser310, Asp534	Met378, Ala312, Ala316, Asp315
	**8**	− 8.9	Gly285, Ala316, Ser289, Lys288, Glu375, Gln537	Ala312, Lys320, Gln537
	**4**	− 8.9	Gly285, Lys288	Arg443, Arg442, Glu540, Lys320, Ala316, Ala312, Ala313
	**19**	− 8.7	None	Gly538, Glu319, Glu540, Ala316, Ser535, Ala312, Ala313
	**1**	− 8.6	None	Ala312, Cys342, Asp315, Ala316,His311
	**22**	− 8.6	Asn459	Phe437, Lys460, Pro434, Gly433, Lys430, Pro402, Tyr457, Ala403
	**23**	− 8.5	Lys430, Gln281, Val456, Tyr457	Phe437, Pro434, Lys430, Leu455
	**24**	− 8.4	Leu417, Asn557, Asn516	Phe422, Pro406, Pro408

Meanwhile, the top-ranked ligands against helicase had binding affinities of − 8.4 to − 9.2 kcal/mol in which the biflavonoids rhusflavanone (**13**) and morelloflavone (**14**) exhibited the strongest affinity (Fig. 3B). Compound **13** occupied the helicase Rec1A domain, which is a component of the nucleotide binding site, through hydrogen bonding of its chromanone (ring A′) hydroxyl and pyrone (ring C′) oxygen with Lys288 (4.76 Å) and Ala316 (3.32 Å), respectively [35]; *pi*-alkyl interactions of its chromene moieties, rings A with Ala316 with Ala316, and ring A′ with Lys320; and *pi*-cation interaction of its hydroxyphenyl moiety (ring B) with Lys320.

Compound **13** also occupied the Rec2A domain of the nucleotide binding site through a hydrogen bond of its pyrone (ring C) carbonyl with Arg443 (4.73 Å), an amide-*pi* stacked interaction of its hydroxyphenyl moiety (ring B′) with Gly538, and a *pi*-sigma interaction of ring B′ with Ser539. A pyrone (ring C) carbonyl further contributed to the binding affinity of compound **13** by binding to Thr286 through van der Waals forces. On the other hand, the dihydroxyphenyl moiety (ring B′) of compound **14** bound Glu341 through a relatively strong hydrogen bonding (3.02 Å) and both Ala312 and Val340 by *pi*-alkyl interactions. Ring A of its chromanone functionality bound Ala313 through a *pi*-alkyl interaction and also Ala312 by *pi*-sigma interaction. These residues are members of the helicase Rec2A domain of the nucleotide binding site. In addition, a benzophyrone hydroxyl (ring A′) of compound **14** bound Asp534 (5.32 Å), which is a residue of the Rec1A helicase domain of the nucleotide binding site.

### Molecular docking with enzymes functioning in the evasion of host immunity

#### SAM-dependent 2′-*O*-methyltransferase complex enzymes (nsp16-nsp10 complex)

**Fig. 4:**
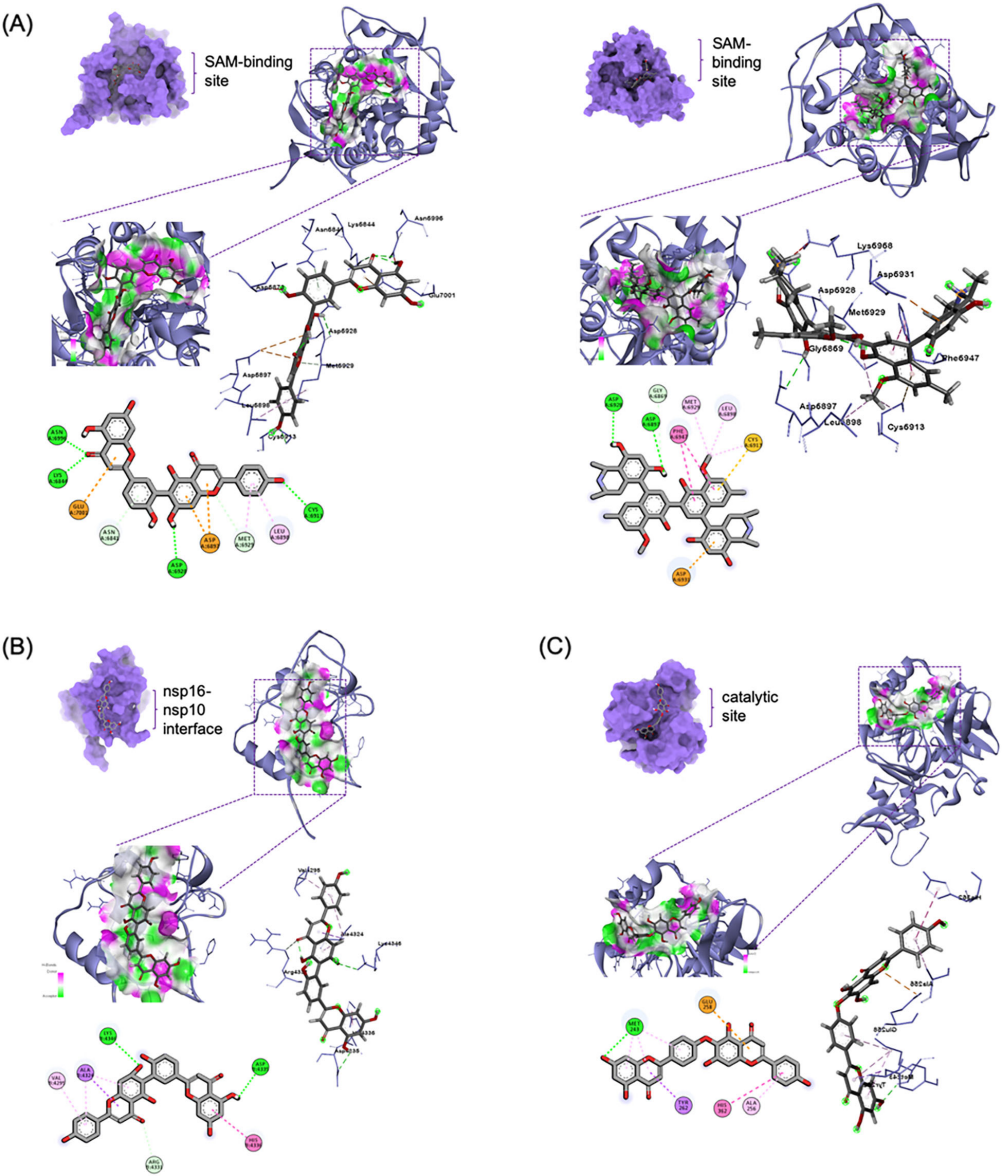
Top 1-binding compounds in complex with nsps involved in host immunity evasion: **A** robustaflavone (**4**) (left) and michellamine B (**19**) (right) complexed with *S*-adenosylmethionine-dependent 2′-*O-*methyltransferase, **B** robustaflavone (**4**) bound to nsp10, and **C** hinokiflavone (**15**) bound to nsp15

Top compounds against nsp16 exhibited affinities from − 9.3 to − 10.6 kcal/mol. The SAM-binding site was targeted and the biflavonoid robustaflavone (**4**) and the alkaloid michellamine B (**19**) demonstrated the greatest affinity (Table 3; Fig. 4A). Hydroxyphenyl ring B of compound **4** exhibited *pi*-alkyl interactions with Leu6898 and Met6929, and a hydrogen bonding with Cys6913 (3.64 Å). The hydroxyphenyl ring B′ showed carbon-hydrogen bonding with Asn6841. Moreover, its benzopyrone moiety (rings A and C) was in *pi*-anion interaction with Asp6897 and its chromene hydrogen was in hydrogen bonding with Asp6928 (3.40 Å). A van der Waals force interaction between its pyrone ring C oxygen and Gly6869 was also observed. Another pyrone moiety (ring C′) also interacted with nsp16 through a *pi*-anion interaction with Glu7001. Lys6844 (5.74 Å) and Asn6996 (4.96 Å) were occupied by the pyrone ring C′ carbonyl through hydrogen bonding. On the other hand, compound **19**’s isoquinoline moiety was in H-bonding with Asp6928 (3.68 Å) and Asp6897 (4.60 Å) and in carbon-hydrogen bonding with Gly6869. Another isoquinoline moiety was in *pi*-anion interaction with Asp6931. Moreover, the naphthalene moiety participated in *pi*-*pi* T-shaped interaction with Phe6947 and in *pi*-sulfur interaction with Cys6914. The methyl group connected to naphthalene manifested alkyl interactions with Met6929, Leu6898, and Cys6913.

**Table 3:** Binding affinities and interactions of top ten ligands against the nsps of the SAM-dependent 2′-O-methyltransferase complex

Target	Cpd	Binding affinity (kcal/mol)	Hydrogen bonds	Other interactions
nsp16	**19**	− 10.6	Asp6897, Asp6928	Cys6913, Cys6914, Met6929, Asp6931, Phe6947, Gly6869, Leu6898
	**4**	− 10.6	Lys6844, Cys6913, Asp6928, Asp6928, Asn6996	Asn6841, Asp6897, Gly6869, Met6929, Leu6898, Glu7001
	**1**	− 10.2	Asn6841, Asp6897, Leu6898, Asp6912	Pro6932, Asp6897, Leu6898, Met6929, Phe6947
	**23**	− 10.2	Asp6931, Cys6913, Tyr6930	Asp6931, Phe6947, Asp6912, Leu6898, Met6929, Asp6897, Gly6869, Asp6928
	**25**	− 9.5	Gly6911, Asp6873, Gly6871, Tyr6930	Leu6898, Cys6913, Met6929, Tyr6930,
	**3**	− 9.5	Asn6841, Lys6844, Asn6996	Met6839, Met6840, Tyr6930, Pro6932, Ser6999
	**18**	− 9.5	Asn6841, Asp6897, Asn6899, Tyr6930, Asn6996, Ser6999, Glu7001	Lys6844, Lys6968
	**13**	− 9.5	Ser759, Asp761	Leu758, Ala688, Asp760, Cys813
	**26**	− 9.4	Asn6899, Asp6873, Lys6844, Asn6841, Asp6928, Leu6898, Asp6912	Cys6913, Phe6947, Gly6869, Tyr6930, Asp6897,
	**20**	− 9.3	Lys6844, Gly6869, Asp6873	Lys6935
nsp10	**4**	− 7.7	Asp4335	Arg4331, Ile4334, Lys4346
	**1**	− 7.4	His4333, Ile4334	Arg4331
	**27**	− 7.3	Asp4344, Leu4345	Tyr4329, Cys4327, His4336, Pro4337, Leu4345, Leu4365
	**15**	− 7.3	Arg4331, His4333, Lys4348, Gly4323, Tyr4349	Val4295, Gly4322, Ala4324
	**19**	− 7.2	Lys4346	Cys4330, His4333, Ala4324, Lys4346, Val4295
	**11**	− 7.2	Asn4293	Cys4294, Lys4296, Val4295, Leu4298
	**23**	− 7.1	Cys4330, His4333, Ile4334, Asp4335, His4336	Lys4346
	**25**	− 7	Tyr4329, His4333, Ala4324, Leu4345, Lys4348	Lys4346, Tyr4349
	**8**	− 7	Cys4343	Lys4346, Gly4347, Phe4342
	**26**	− 6.9	Leu4345, Lys4348, Gly4347, Ala4324, His4333, Ile4334, Tyr4329	Lys4348, Arg4331, Lys4346,
	**17**	− 6.9	Tyr4329, His4336	Asn4293, Arg4331, Ala4324, His4333, Lys4346, Ile4334
	**9**	− 6.9	Ala4324, Lys4346, Lys4348, Tyr4349	Val4295, Gly4322, Gly4347, Ala4324, Tyr4349
	**20**	− 6.9	Leu4345, Gly4347, Lys4348	Ala4324, Arg4331, His4333

In connection, top compounds against nsp10 showed binding affinities of − 6.9 to − 7.7 kcal/mol. The interface between nsp10 and nsp16 was targeted and several interactions were observed. Biflavonoid robustaflavone (**4**) had the highest affinity (Fig. 4B). Its pyrone ring C′ was in carbon-hydrogen bonding with Ile4334. Chromanone (ring A′) hydroxyl formed a strong H-bonding with Asp4335 (3.50 Å). Carbon atoms of chromanone (rings A and C) and hydroxyphenyl ring B′ formed salt bridges with Lys4346 while ring C′ carbonyl exhibited a salt bridge with Arg4331.

#### Endoribonuclease (nsp15)

Top-scoring compounds against nsp15 exhibited affinities of − 8.6 to − 7.3 kcal/mol, stronger (Table 4). The biflavonone hinokiflavone (**15**) scored the highest affinity, noting its interactions with its putative binding site that is proximal to the catalytic triad of His235, His250, and Lys290: flavone moiety (rings A, B, and C) in hydrogen bonding (4.55 Å) and *pi*-alkyl interaction with Met243, pyrone ring C in *pi*-sigma interaction with Tyr262, pyrone ring C′ in *pi*-anion interaction with Glu258, and hydroxyphenyl ring B′ in *pi*-alkyl and *pi*-*pi* stacked interactions with Ala256 and His362, respectively (Fig. 4C).

**Table 4:** Binding affinities and interactions of top ten ligands against nsp15

Target	Cpd	Binding affinity (kcal/mol)	Hydrogen bonds	Other interactions
nsp15	**15**	− 8.6	Met243	Met243, Tyr262, Glu258, His362, Ala256
	**4**	− 8.5	None	Lys281, Glu285, Tyr262, Met243
	**1**	− 8.4	Gly254	Met243, Ala256, Glu258
	**25**	− 8.1	Phe265, Ser266	Lys281, Ser266, Ala256
	**23**	− 8	None	Glu258, Ala256, Gly263, Asp264, Phe265
	**26**	− 7.8	Glu285, Glu364	Ala256, Ser266, Met243, Lys281, Glu285
	**12**	− 7.7	Glu285	Ala256, Met243, Tyr262, Lys281
	**14**	− 7.7	Arg282	Glu285, Lys281, Phe265
	**13**	− 7.3	Gly263, Asp264	Ala256, His259, Asp264, Met243, Glu285
	**17**	− 7.3	Glu258	None

### Druggability, ADME, and toxicity

Six of the 20 top-scoring and multi-targeting repurposed phytochemicals were found to be druggable according to Lipinski’s rule of five (Table 5). Hinokiflavone (**15**) is a top-scoring, multi-targeting, druggable compound. Moreover, compounds **5** and **17** were multi-targeting and exhibited good gastrointestinal absorption properties.

**Table 5:** Druggability of top, multi-targeting compounds according to Lipinski’s rule of five

Cpd	MW <500	#H-bond acceptors <10	#H-bond donors <5	Lipophilicity MLogP<5	Lipinski violations	Drug-likeness	Target
**1**	538.46	10	6	0.25	2	No	PLpro, 3CLpro, RdRp, helicase, nsp10, nsp16, nsp15
**2**	426.72	1	0	6.92	1	Yes	PLpro, RdRp
**3**	570.8	4	0	5.03	2	No	PLpro, 3CLpro, RdRp, nsp16
**4**	538.46	10	6	0.25	2	No	PLpro, 3CLpro, RdRp, helicase, nsp10, nsp16, nsp15
**5**	472.7	4	3	4.97	1	Yes	PLpro, RdRp
**8**	538.46	10	6	0.25	2	No	PLpro, 3CLpro, helicase, nsp10
**9**	782.53	22	13	− 2.56	3	No	PLpro, RdRp, nsp10
**10**	456.7	3	2	5.82	1	Yes	PLpro, RdRp
**11**	540.47	10	3	0.41	2	No	3CLpro, nsp10
**12**	498.74	4	1	5.97	1	Yes	3CLpro, nsp15
**13**	542.49	10	6	0.58	2	No	3CLpro, helicase, nsp16, nsp15
**14**	556.47	11	7	− 0.08	3	No	3CLpro, helicase, nsp15
**15**	538.46	10	5	0.52	1	Yes	3CLpro, RdRp, helicase, nsp10, nsp15
**17**	520.65	8	4	1.95	1	Yes	3CLpro, nsp10, nsp15
**18**	992.64	28	14	− 3.39	3	No	RdRp, np16
**19**	756.88	10	8	3.18	2	No	RdRp, helicase, nsp10, nsp16
**20**	1008.75	28	14	− 3.39	3	No	RdRp, nsp10, nsp16
**23**	578.52	14	8	− 2.96	3	No	helicase, nsp10, nsp16, nsp15
**25**	516.45	12	7	− 0.35	3	No	nsp10, nsp16, nsp15
**26**	516.45	12	7	− 0.35	3	No	nsp10, nsp16, nsp15

In addition, compounds **25** and **26** showed the best solubility in water of − 2.85, thereby depicting good excretion properties (Table 6). Toxicity prediction through OSIRIS Property Explorer showed that all top compounds except **11**, **14**, **15**, **18**, **19**, and **20** have no mutagenic, tumorigenic, irritant, and reproductive toxicity risks (Table 6).

**Table 6:** Toxicity risks of top, multi-targeting compounds as predicted by OSIRIS Property Explorer

Cpd	Toxicity risk				Solubility (LogS)
	Mutagenic	Tumorigenic	Irritant	Reproductive effective	
**1**	No	No	No	No	− 6.16
**2**	No	No	No	No	− 6.97
**3**	No	No	No	No	− 7.66
**4**	No	No	No	No	− 6.18
**5**	No	No	No	No	− 5.66
**8**	No	No	No	No	− 6.18
**9**	No	No	No	No	− 5.89
**10**	No	No	No	No	− 6.11
**11**	No	No	No	High Risk	− 5.11
**12**	No	No	No	No	− 6.37
**13**	No	No	No	No	− 5.75
**14**	No	No	No	High Risk	− 4.82
**15**	No	No	No	High Risk	− 6.69
**17**	No	No	No	No	− 4.42
**18**	No	No	High Risk	No	− 3.54
**19**	No	High Risk	No	No	− 11.38
**20**	No	No	High Risk	No	− 3.54
**23**	No	No	No	No	− 2.95
**25**	No	No	No	No	− 2.85
**26**	No	No	No	No	− 2.85

### Molecular dynamics simulations

**Fig. 5:**
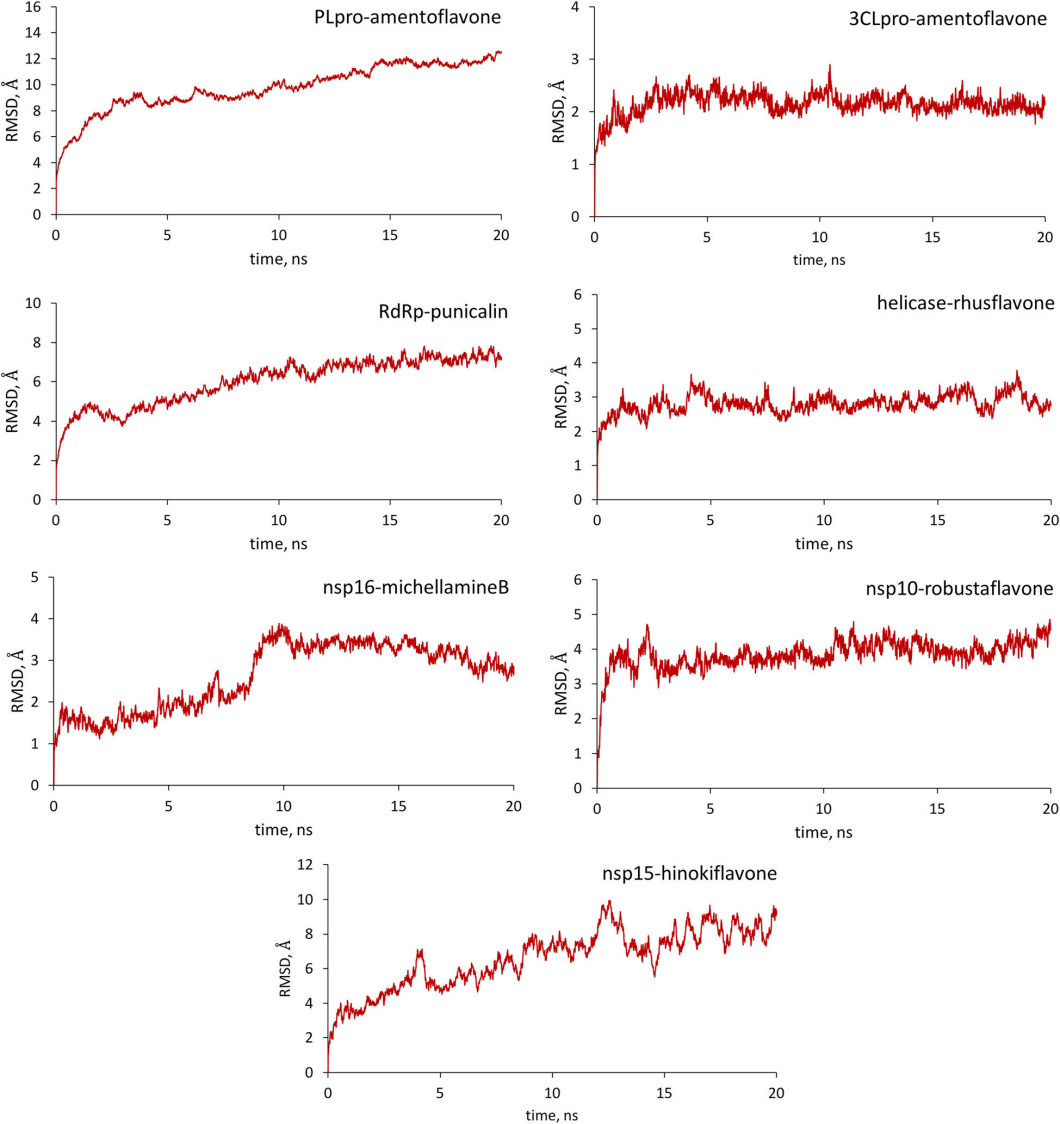
Root mean square deviation (RMSD, Å) as a function of time of the top-binding complexes

Molecular dynamics (MD) simulation was performed on the top-binding ligands, chosen based on molecular docking and ADMET analyses, to assess at an atomic level the binding behavior of the various polyphenols against SARS-CoV-2 non-structural proteins. The stability of the complexes, specifically PLpro-amentoflavone (**1**), 3CLpro-amentoflavone (**1**), RdRp-punicalin (**9**), helicase-rhusflavanone (**13**), nsp16-michellamine B (**19**), nsp10-robustaflavone (**4**), and nsp15-hinokiflavone (**15**), was evaluated using post-simulation parameters root mean square deviation (RMSD) and root mean square fluctuations (RMSF). RMSD is one of the widely used analyses using MD trajectories of protein-ligand complexes to establish equilibrium within a given simulation period. Based on RMSD analysis which was measured as an average throughout a 20-ns simulation, the complexes attained dynamic stability (Fig. 5). In the case of the amentoflavone (**1**)-bound PLpro, it took some time for the complex to reach equilibrium. As shown in the plot of RMSD (Å) versus simulation time (ns), a steady increase in RMSD can be observed up to 15 ns before stabilization occurs. A similar trend can be observed for the RdRp-punicalin (**9**) complex. After a steady rise in RMSD, the complex achieved equilibrium around 13 ns with an average RMSD value of 7.1 Å. Among the complexes, the amentoflavone (**1**)-bound 3CLpro appeared to be the most stable complex having the lowest average equilibrium RMSD (2.2 Å). Although a minor fluctuation can be observed around 11 ns, the complex remained stable for the entire simulation time. In the case of nsp16-michellamine B (**19**) complex, an incremental increase in RMSD can be noted from the start of the simulation until 8 ns and a relatively high divergence can be seen around 9 ns. However, the system gained equilibrium thereafter. For the helicase-rhusflavanone (**13**) complex, several minor fluctuations can be noticed from initial binding stage up to mid simulation time. Despite this observation, the average RMSD of the complex remained low (2.8 Å). Another relatively stable complex is robustaflavone (**4**) bound to nsp10 with an average equilibrium RMSD of 3.9 Å. Shortly after ligand-binding, the complex attained equilibrium. Lastly, the nsp15-hinokiflavone (**15**) complex exhibited relatively low stability based on the RMSD plot where some minor fluctuations are noted at the beginning of the simulation up to 15 ns.

**Fig. 6:**
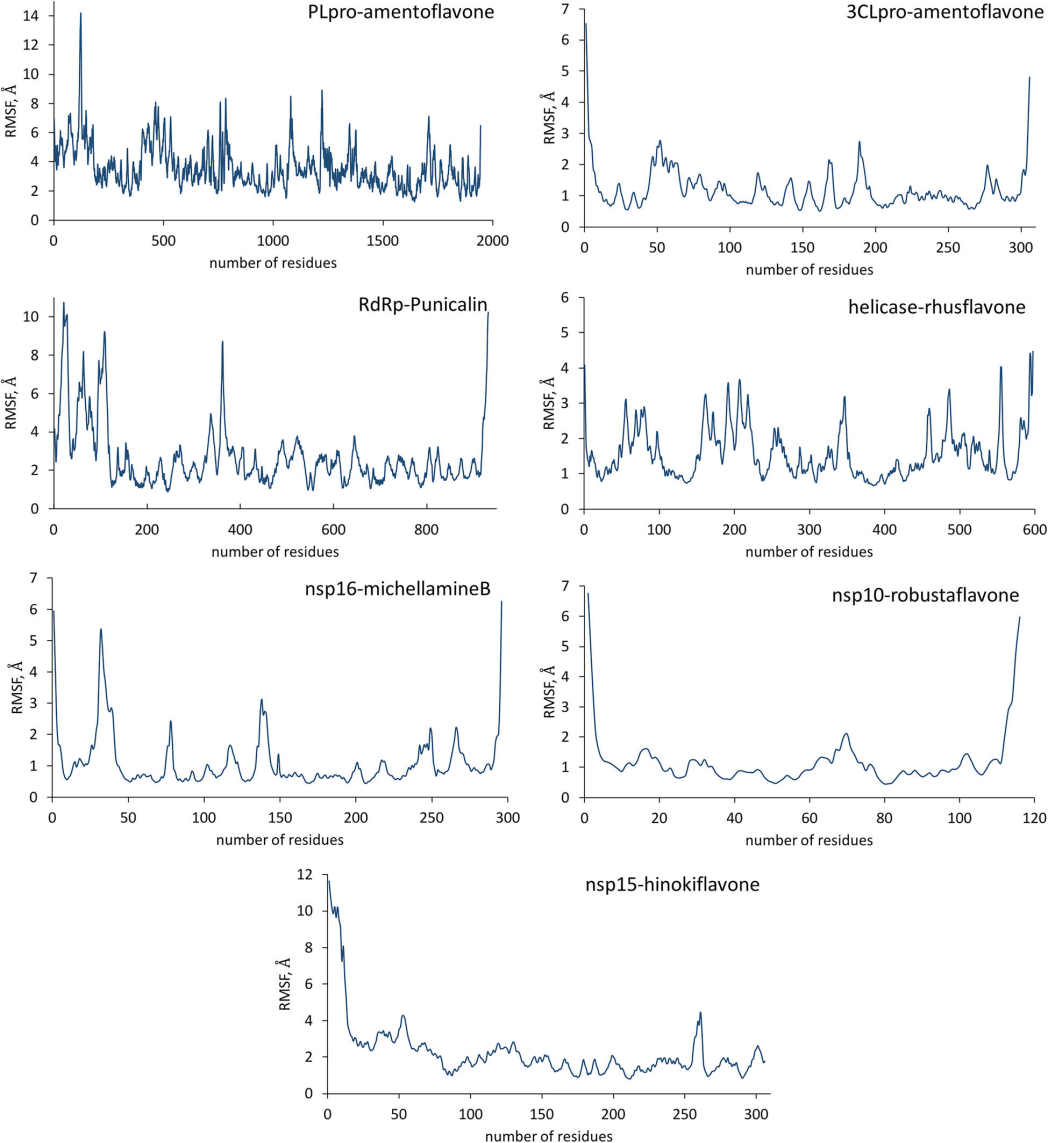
Time-averaged root mean square fluctuations (RMSF, Å) as a function amino acid residue sequence number of the top-binding complexes

The time-averaged residual fluctuations of the seven top-binding complexes were also analyzed on the basis of trajectory data within a 20-ns simulation (Fig. 6). The results of the residual RMSF analysis revealed that most of the complexes, particularly 3CLpro-amentoflavone (**1**), nsp16-michellamine B (**19**), nsp10-robustaflavone (**4**), helicase-rhusflavanone (**13**), have average RMSF values ranging from 1.1 to 1.6 Å and have shown relatively stable fluctuation patterns. These data are consistent with RMSD analysis, which confirm that the said complexes are stable. For the hinokiflavone (**15**)-bound nsp15 complex, the average residual fluctuations are slightly higher (2.2 Å). For the larger protein complexes—RdRP and PLpro, higher fluctuations were observed, averaging 3.3 Å and 3.5 Å, respectively. Such an RMSF pattern for the two complexes justifies the observed longer time for stabilization to occur during RSMD calculations.

## Discussion

**Fig. 7:**
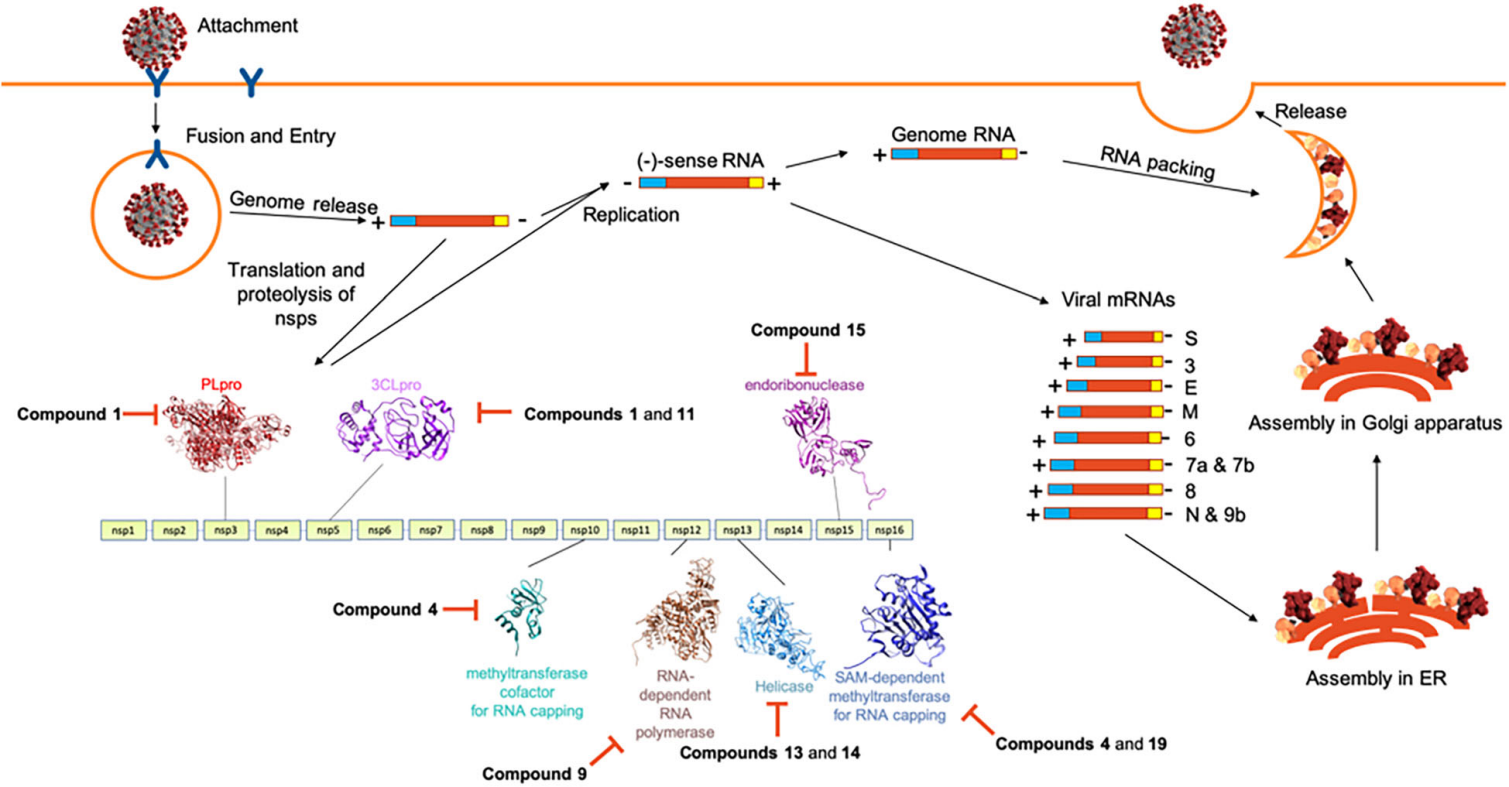
The SARS-CoV-2 life cycle highlighting the role of nsps in replication and transcription and the potential inhibited target SARS-CoV-2 nsps of repurposed anti-HIV RT phytochemicals. SARS-CoV-2 virion image credit: CDC/ Alissa Eckert (MSMI) and Dan Higgins (MAMS)

The SARS-CoV-2 non-structural proteins (nsps) play vital roles in the virus’ pathogenesis, survival, and virulence. A number of these nsps have been considered as attractive and important drug targets due to their involvement in viral post-translational processing, replication, and host immunity evasion mechanisms (Fig. 7). The cysteine proteases, nsp3 (PLpro) and nsp5 (3CLpro), are involved in the autolytic cleavage of the polyproteins pp1a and pp1ab wherein PLpro cleaves 3 sites at the *N*-terminus while 3CLpro cleaves through the remaining sites (11 sites in pp1ab) to release the nsps [36]. Proceeding to the replication-transcription complex, nsp12 (RdRp) elongates the daughter strand through the polymerization of nucleotides while nsp13 (helicase) clears RNA secondary structures and RNA-binding proteins [37]. The nsp 16 (SAM-dependent 2′-*O*-methyltransferase) in complex with nsp10 as its cofactor provides a 5′ cap to the RNA genome through C2′-*O*-methyl-ribosyladenine, conferring RNA stability and host cell immunity protection [38]. Lastly, the nsp15 (endoribonuclease) hinders recognition of dsRNA intermediates by host dsRNA sensors [33]. Our results, therefore, highlight the role of anti-HIV RT phytochemicals as potential antagonists of SARS-CoV-2 by interfering with the discussed mechanisms.

Natural products have been a subject of investigation concerning their ability to antagonize SARS-CoV-2 due to their availability and wide range of health benefits [34, 35, 39–41]. In relation, repurposing established anti-HIV phytochemicals means that the lead compounds in this study can be easily obtained from previously explored plants that are consumed by populations. Here, we focused on the employment of computational target-based drug discovery methodologies, such as molecular docking, molecular dynamic simulations, and pharmacokinetic property predictions in search for potential hits for inhibiting the aforementioned SARS-CoV-2 nsps. Our study revealed that the biflavonoid amentoflavone (**1**) showed the highest binding to both SARS-CoV-2 cysteine proteases PLpro and 3CLpro. Compound **1**, isolated from the Chinese olive fruit, *Canarium album* [42], was also reported in a previous study to be a potent inhibitor of SARS-CoV PLpro [43, 44]. Volkensiflavone (**11**) from the seeds and rinds of *Garcinia intermedia* [45] was another top compound against 3CLpro. Punicalin (**9**) from the pomegranate *Punica granatum* peel [46] exhibited high binding propensity against RdRp, an enzyme considered to be a promising target inhibiting viral replication. Morelloflavone (**14**) from *G*. *intermedia* was first to be reported here with an inhibitory potential against SARS-CoV-2 helicase. Interestingly, its potential extends against 3CLpro [47]. Robustaflavone (**4**) from the leaves of *Garcinia epunctata* [48] showed the best potential against the 2′-*O*-methyltransferase and its cofactor. This is the first investigation of its activity against these nsps aside from its interaction with 3CLpro [49]. On the other hand, michellamine B (**19**) from *Ancistrocladus korupensis* leaves [50] manifested an inhibitory potential against nsp16, therefore opening the doors of phenolic alkaloids against SARS-CoV-2. Lastly, hinokiflavone (**15**) from *Selaginella tamariscina* [51] was reported to be a potential 3CLpro inhibitor and potent against the replication-transcription complex [43, 47, 49, 52]. This, however, is the first investigation of its activity against the endoribonuclease of SARS-CoV-2 in silico. The multi-targeting potential of some of these compounds increases the chance of getting a maximal inhibitory effect [53].

To further validate the molecular docking analysis, the top-binding ligands were submitted for molecular dynamic simulations. Through post-simulation analyses, the top-binding ligands were generally found to be dynamically stable upon binding to respective proteins. Although most of the top 1 compounds were predicted in silico to be non-druggable, efforts are rising to explore compounds in the oral druggable space beyond the rule of five (bRo5) [54, 55]. Additionally, four of these did not manifest toxicity in silico. The biflavonoids volkensiflavone (**11**), morelloflavone (**14**), and hinokiflavone (**15**) were computationally predicted as non-mutagenic, non-tumorigenic, and non-irritant, but were predicted to pose reproductive toxicity risk which may be attributed to their chromene and hydroxyphenyl moieties. It should be noted, however, that hinokiflavone (**15**) is a druggable top 1 compound. Michellamine B (**19**) was predicted to be tumorigenic due to its naphthalene moiety. In addition, compounds **5** and **17** exhibited good gastrointestinal absorptive features as implicated by their favorable lipophilicity and polar surface area [56]. These also did not manifest any form of toxicity in silico. Despite computational incompatibilities, these compounds can still serve as templates for drug design and undergo in vitro and in vivo assays for validating their anti-SARS-CoV-2 properties, noting that their promising polyphenolic nature allowed them to form hydrogen bonds with key residues of the SARS-CoV-2 nsps. With the validation of pre-clinical experiments, the secondary metabolites can be produced through in vitro plant tissue cultures that can be augmented by metabolic engineering, elicitation, and even the use of bioreactors [57, 58].

## Conclusions

The search for anti-COVID-19 therapeutic agents is a response to the continuous spread of the virus amidst vaccine availability. The similarity between the pathogenesis of HIV and SARS-CoV-2 inspired the repurposing of previously reported anti-HIV reverse transcriptase phytochemicals against SARS-CoV-2 nsps implicated in viral replication, post-translational processing, and host immunity evasion mechanisms. The top-ranking polyphenolics amentoflavone (**1**), robustaflavone (**4**), punicalin (**9**), volkensiflavone (**11**), rhusflavanone (**13**), morelloflavone (**14**), hinokiflavone (**15**), and michellamine B (**19**) can be further screened using confirmatory in vitro and in vivo assays, and can serve as prototypes for designing novel anti-COVID-19 drugs in consideration of their polyphenolic nature. As promising drug templates, functionalities in the compound structure can be modified to improve druggability and pharmacokinetic properties.

## Additional files

Additional file 1

## Data Availability

All data generated in this study are included in this published article and the supplementary information files.

## Abbreviations

3CLpro: 3-Chymotrypsin-like protease
ADME: Absorption, digestion, metabolism, and excretion
BE: Binding energy
COVID-19: Coronavirus disease 2019
Cpd: Compound
dsDNA: Double-stranded DNA
HIV: Human immunodeficiency virus
kcal/mol: Kilocalorie per mole
nsp: Non-structural protein
PDB: Protein data bank
PLpro: Papain-like protease
RdRp: RNA-dependent RNA polymerase
RNA: Ribonucleic acid
RT: Reverse transcriptase
SARS-CoV-2: Severe acute respiratory syndrome coronavirus 2
