# Supplementary material for: Anti-HIV reverse transcriptase plant polyphenolic natural products with in silico inhibitory properties on seven non-structural proteins vital in SARS-CoV-2 pathogenesis
Source: J Genet Eng Biotechnol. 2021 Jul 16;19:104. doi: 10.1186/s43141-021-00206-2 (PMC8284420; doi:10.1186/s43141-021-00206-2)
Supplement: Supplementary file 1 — Additional file 1 [file 43141_2021_206_MOESM1_ESM.docx]

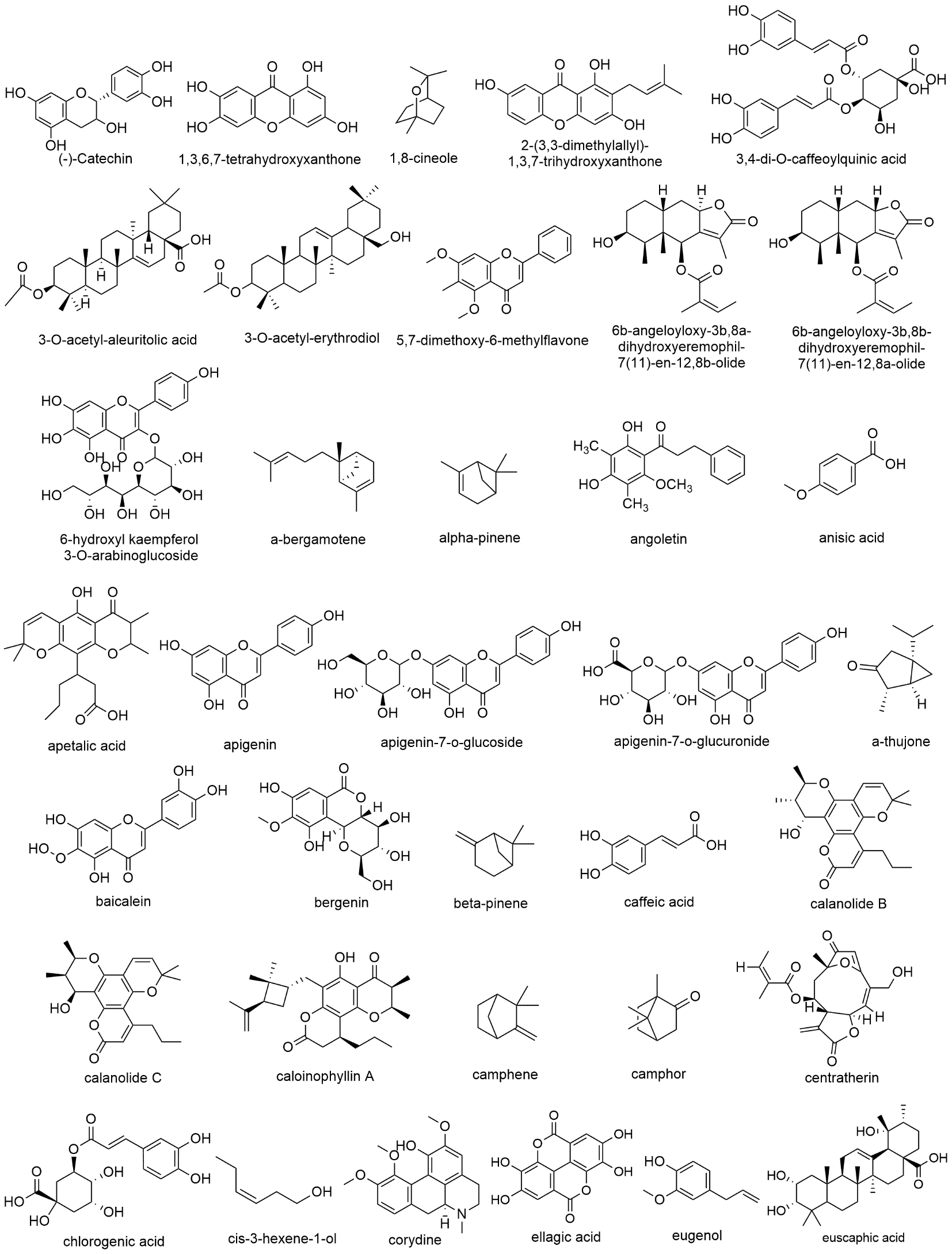


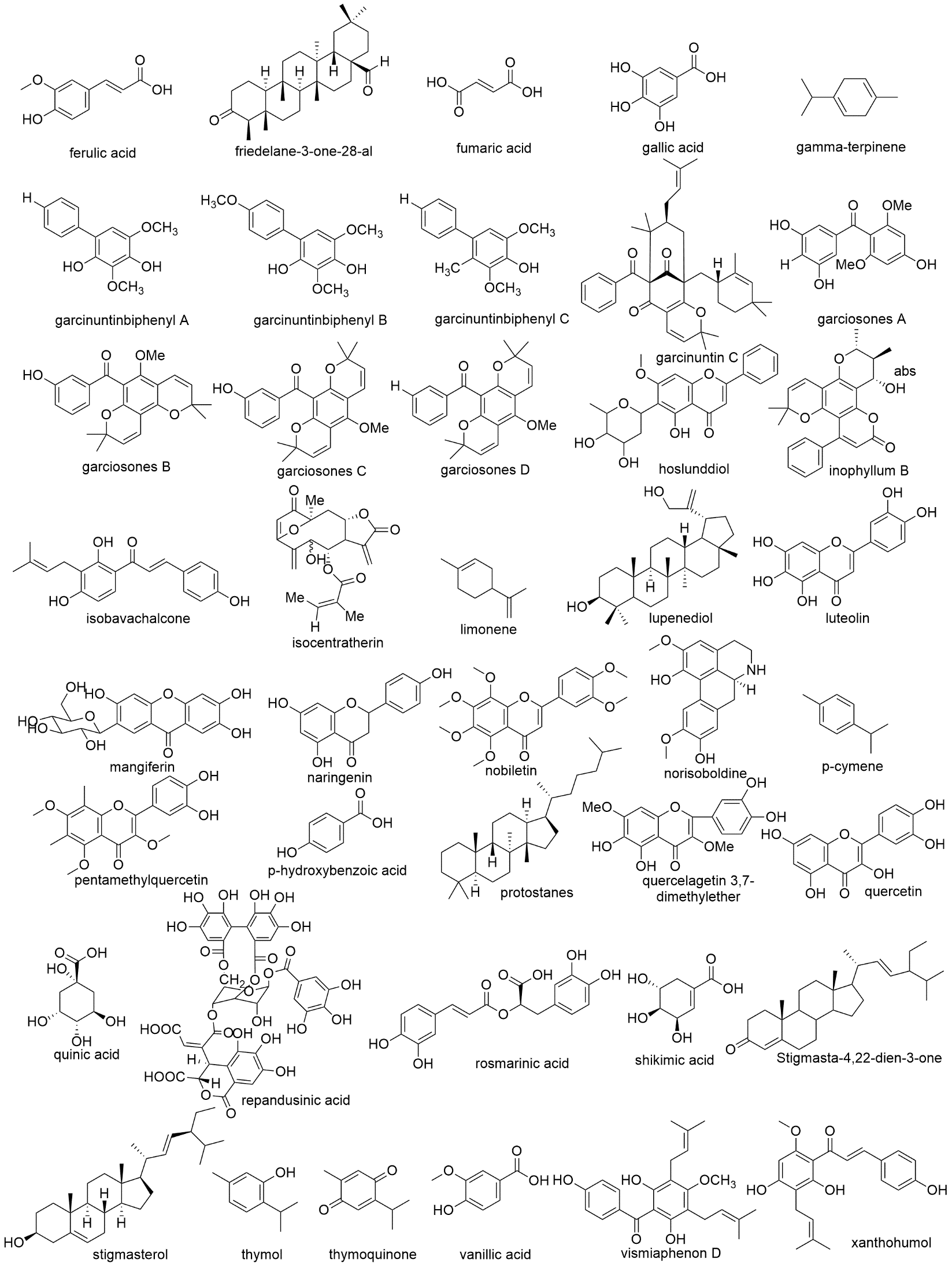


Supplementary Figure 1 Remaining 77 anti-HIV RT compounds tested against SARS-CoV-2 nsps: PLpro, 3CLpro, RdRp, helicase, SAM-dependent 2’-*O*-methyltransferase and its cofactor (nsp10), and endoribonuclease.

Supplementary Table 1 Binding affinities (kcal/mol) of 104 anti-HIV RT phytochemicals to SARS-CoV-2 nsps.

| **Ligand** | **PLPro** | **3CLpro** | **RdRp** | **Helicase** | **nsp16** | **nsp10** | **nsp15** |
| --- | --- | --- | --- | --- | --- | --- | --- |
| (-)-Catechin | -8.0 | -7.0 | -6.8 | -7.0 | -8.1 | -5.9 |  |
| 1,3,6,7-tetrahydroxyxanthone | -7.3 | -6.8 | -6.9 | -6.7 | -7.1 | -5.7 |  |
| 1,5-dicaffeoylquinic acid | -8.6 | -6.9 | -7.3 | -7.6 | -9.4 | -6.9 | -7.8 |
| 1,8-cineole | -5.7 | -4.1 | -5.0 | -4.7 | -4.8 | -5.1 |  |
| 2-(3,3-dimethylallyl)-1,3,7-trihydroxyxanthone | -8.0 | -6.8 | -7.2 | -7.0 | -7.7 | -5.8 |  |
| 3,4-dicaffeoylquinic acid | -8.2 | -7.9 | -6.6 | -7.2 | -8.2 | -6.0 | -5.8 |
| 3,4-di-O-caffeoylquinic acid | -9.1 | -7.7 | -7.5 | -6.8 | -9.2 | -6.1 |  |
| 3,5-dicaffeoylquinic acid | -8.5 | -7.5 | -7.5 | -7.3 | -9.5 | -7.0 | -8.1 |
| 3-O-acetyl-aleuritolic acid | -9.3 | -7.0 | -8.5 | -7.8 | -8.0 | -6.2 |  |
| 3-O-acetyl-erythrodiol | -9.6 | -6.9 | -8.4 | -7.9 | -8.1 | -6.6 |  |
| 5,7-dimethoxy-6-methylflavone | -7.7 | -6.6 | -6.3 | -7.1 | -7.4 | -5.3 |  |
| 6b-angeloyloxy-3b,8a-dihydroxyeremophil-7(11)-en-12,8b-olide | -8.6 | -6.7 | -7.2 | -7.0 | -7.6 | -5.3 |  |
| 6b-angeloyloxy-3b,8b-dihydroxyeremophil-7(11)-en-12,8a-olide | -8.3 | -6.6 | -6.9 | -7.1 | -7.1 | -5.0 |  |
| 6-hydroxyl kaempferol 3-O-arabinoglucoside | -8.7 | -7.8 | -7.1 | -8.3 | -7.5 | -5.6 |  |
| a-bergamotene | -7.0 | -5.0 | -5.6 | -6.5 | -5.7 | -6.1 |  |
| agathisflavone | -10.2 | -8.4 | -8.4 | -8.9 | -9.1 | -7.0 | -6.8 |
| alpha-pinene | -5.6 | -3.8 | -4.7 | -4.7 | -4.8 | -5.1 |  |
| amentoflavone | -10.8 | -8.6 | -8.6 | -8.6 | -10.2 | -7.4 | -8.4 |
| angoletin | -7.2 | -6.5 | -6.2 | -6.9 | -7.2 | -6.4 |  |
| anisic acid | -5.3 | -5.1 | -4.9 | -5.1 | -6.1 | -4.8 |  |
| apetalic acid | -8.2 | -6.6 | -7.5 | -6.6 | -6.8 | -5.5 |  |
| apigenin | -7.9 | -7.1 | -6.6 | -6.7 | -7.9 | -5.6 |  |
| apigenin-7-o-glucoside | -8.6 | -7.2 | -7.7 | -7.8 | -8.6 | -6.3 |  |
| apigenin-7-o-glucuronide | -8.6 | -7.6 | -7.7 | -7.5 | -8.9 | -6.5 |  |
| apigenin-7-o-rutinoside | -9.5 | -7.8 | -8.2 | -8.5 | -10.2 | -7.1 | -8.0 |
| artemisinin | -8.7 | -6.2 | -7.1 | -6.9 | -6.6 | -7.3 | -6.2 |
| a-thujone | -5.4 | -4.3 | -5.1 | -5.1 | -5.1 | -5.1 |  |
| Baicalein | -8.4 | -7.4 | -6.9 | -6.9 | -8.4 | -5.9 |  |
| bergenin | -7.1 | -6.3 | -6.8 | -6.5 | -7.0 | -5.5 |  |
| beta-pinene | -5.7 | -3.8 | -4.8 | -5.3 | -4.7 | -5.4 |  |
| betulinic acid | -10.2 | -7.0 | -7.9 | -6.6 | -7.5 | -5.7 | -6.6 |
| caffeic acid | -6.2 | -5.1 | -5.1 | -6.0 | -6.5 | -4.9 |  |
| calanolide B | -9.2 | -7.1 | -7.4 | -7.5 | -8.6 | -5.6 |  |
| calanolide C | -8.8 | -6.8 | -7.6 | -7.4 | -8.1 | -5.4 |  |
| caloinophyllin A | -9.1 | -7.1 | -7.3 | -7.3 | -8.1 | -5.9 |  |
| camphene | -5.4 | -3.7 | -4.7 | -4.7 | -5.1 | -5.2 |  |
| camphor | -5.8 | -4.1 | -4.9 | -4.7 | -4.3 | -5.5 |  |
| centratherin | -8.1 | -7.3 | -6.8 | -7.4 | -7.9 | -5.5 |  |
| chlorogenic acid | -8.2 | -7.2 | -6.3 | -7.2 | -7.5 | -5.6 |  |
| cis-3-hexene-1-ol | -4.6 | -3.6 | -3.4 | -3.9 | -3.7 | -3.8 |  |
| corydine | -6.9 | -6.8 | -6.3 | -6.6 | -6.9 | -5.4 |  |
| digitoxigenin-3-O-glucoside | -9.6 | -7.9 | -8.1 | -8.2 | -9.1 | -6.9 | -7.3 |
| ellagic acid | -8.3 | -6.7 | -7.6 | -6.9 | -7.6 | -6.1 |  |
| eugenol | -5.5 | -4.6 | -5.1 | -5.3 | -5.6 | -5.6 |  |
| euscaphic acid | -10.0 | -7.5 | -8.2 | -7.7 | -8.2 | -6.3 |  |
| ferulic acid | -5.8 | -5.4 | -5.2 | -6.1 | -6.7 | -4.5 |  |
| friedelane-3-one-28-al | -10.0 | -7.0 | -8.0 | -8.0 | -7.8 | -6.3 |  |
| friedelin | -10.7 | -7.7 | -8.9 | -7.6 | -8.0 | -6.3 | -6.7 |
| fumaric acid | -4.9 | -4.4 | -3.9 | -5.1 | -4.5 | -4.3 |  |
| gallic acid | -5.8 | -4.9 | -5.1 | -5.4 | -6.0 | -5.4 |  |
| gamma-terpinene | -5.6 | -4.4 | -4.7 | -5.3 | -5.2 | -5.1 |  |
| garcinuntin A | -10.7 | -7.9 | -8.6 | -7.3 | -9.5 | -6.3 | -6.5 |
| garcinuntin B | -9.6 | -7.4 | -8.0 | -8.6 | -8.6 | -5.9 | -6.9 |
| garcinuntin C | -9.6 | -6.8 | -7.2 | -6.5 | -7.8 | -5.4 |  |
| garcinuntinbiphenyl A | -6.8 | -5.9 | -5.9 | -6.1 | -6.7 | -4.8 |  |
| garcinuntinbiphenyl B | -6.9 | -5.8 | -5.8 | -6.3 | -6.8 | -4.7 |  |
| garcinuntinbiphenyl C | -7.3 | -5.9 | -6.0 | -6.3 | -7.1 | -5.2 |  |
| garciosones A | -6.6 | -5.9 | -6.3 | -6.5 | -7.3 | -5.2 |  |
| garciosones B | -9.0 | -7.3 | -7.3 | -7.8 | -8.1 | -6.4 |  |
| garciosones C | -8.5 | -6.9 | -7.2 | -7.6 | -7.8 | -5.7 |  |
| garciosones D | -8.5 | -6.8 | -6.8 | -7.2 | -7.7 | -5.6 |  |
| garcisaterpenes A | -9.1 | -8.5 | -7.6 | -7.4 | -8.0 | -5.6 | -7.7 |
| garcisaterpenes C | -9.1 | -7.1 | -7.7 | -8.4 | -8.9 | -6.4 | -6.9 |
| hinokiflavone | -9.8 | -8.1 | -8.9 | -9.0 | -7.2 | -7.3 | -8.6 |
| hoslunddiol | -9.0 | -7.6 | -7.7 | -7.5 | -8.1 | -5.7 |  |
| inophyllum B | -8.4 | -7.6 | -8.1 | -7.7 | -8.5 | -6.2 |  |
| isobavachalcone | -7.8 | -6.3 | -6.9 | -6.6 | -7.6 | -5.4 |  |
| isocentratherin | -8.2 | -6.2 | -7.7 | -6.9 | -6.9 | -5.3 |  |
| limonene | -5.4 | -4.3 | -4.6 | -5.1 | -5.2 | -5.0 |  |
| lupenediol | -9.6 | -7.3 | -8.3 | -7.3 | -7.3 | -6.0 |  |
| lupenoic acid | -9.7 | -6.9 | -8.7 | -6.6 | -7.4 | -6.3 | -6.5 |
| luteolin | -8.3 | -7.1 | -6.9 | -7.3 | -8.2 | -6.1 |  |
| mangiferin | -9.1 | -7.2 | -7.5 | -7.5 | -8.3 | -6.0 |  |
| michellamine B | -9.9 | -7.6 | -8.8 | -8.7 | -10.6 | -7.2 | -6.3 |
| morelloflavone | -9.7 | -8.4 | -7.4 | -9.2 | -8.6 | -6.0 | -7.7 |
| naringenin | -8.0 | -7.2 | -6.6 | -6.9 | -7.8 | -5.7 |  |
| nobiletin | -7.1 | -6.6 | -6.1 | -6.6 | -8.0 | -5.4 |  |
| norisoboldine | -7.5 | -6.5 | -6.7 | -6.9 | -8.5 | -5.8 |  |
| oleanolic acid | -10.3 | -6.9 | -8.3 | -6.8 | -9.2 | -6.0 | -6.7 |
| p-cymene | -5.6 | -4.3 | -4.6 | -5.5 | -5.0 | -5.2 |  |
| pentamethylquercetin | -8.0 | -6.8 | -7.0 | -6.9 | -7.3 | -5.5 |  |
| p-hydroxybenzoic acid | -5.3 | -4.7 | -4.8 | -5.8 | -6.1 | -5.0 |  |
| pomolic acid | -10.4 | -7.0 | -8.6 | -7.3 | -8.3 | -6.1 | -6.9 |
| protostanes | -8.6 | -5.6 | -7.3 | -7.3 | -6.7 | -5.0 |  |
| punicalin | -10.2 | -7.7 | -9.5 | -7.2 | -8.5 | -6.9 | -0.5 |
| quercelagetin 3,7-dimethylether | -8.3 | -6.9 | -7.1 | -6.7 | -7.6 | -6.2 |  |
| quercetin | -8.2 | -7.4 | -6.9 | -6.8 | -8.4 | -5.8 |  |
| quinic acid | -6.2 | -5.2 | -4.8 | -5.3 | -5.2 | -5.3 |  |
| repandusinic acid | -8.4 | -7.1 | -8.5 | -7.5 | -8.7 | -6.0 |  |
| repandusinic acid A - monopotassium salt | -9.0 | -6.6 | -8.8 | -8.2 | -9.3 | -6.9 | -0.3 |
| repandusinic acid A - monosodium salt | -9.4 | -7.1 | -8.9 | -6.6 | -9.5 | -6.5 | -4.8 |
| rhusflavanone | -9.9 | -8.5 | -8.3 | -9.2 | -9.5 | -6.7 | -7.3 |
| robustaflavone | -10.6 | -8.5 | -8.8 | -8.9 | -10.6 | -7.7 | -8.5 |
| rosmarinic acid | -7.8 | -5.9 | -6.9 | -7.4 | -8.5 | -5.1 |  |
| shikimic acid | -5.8 | -5.0 | -4.7 | -5.4 | -5.7 | -5.5 |  |
| Stigmasta-4,22-dien-3-one | -9.1 | -6.2 | -7.5 | -7.0 | -8.5 | -5.3 |  |
| stigmasterol | -8.7 | -6.0 | -7.1 | -7.4 | -7.8 | -5.6 |  |
| thymol | -5.5 | -4.4 | -5.2 | -6.0 | -5.4 | -5.3 |  |
| thymoquinone | -5.8 | -4.8 | -5.5 | -5.7 | -5.7 | -5.5 |  |
| ursolic acid | -10.1 | -7.1 | -9.1 | -7.2 | -8.3 | -6.4 | -5.9 |
| vanillic acid | -5.7 | -5.1 | -5.0 | -5.4 | -6.5 | -5.1 |  |
| vismiaphenon D | -8.5 | -6.8 | -7.2 | -6.6 | -7.6 | -5.6 |  |
| volkensiflavone | -9.9 | -8.6 | -8.1 | -8.3 | -8.9 | -7.2 | -6.9 |
| xanthohumol | -8.1 | -6.8 | -6.9 | -6.6 | -7.7 | -4.9 |  |

Supplementary Table 2 Grid box parameters encompassing the ligand-binding cavity.

| Enzyme | Center (x, y, z) | Size (x, y, z) |
| --- | --- | --- |
| 3CLpro (6LU7) | 85, 67, 80 | 25, 25, 25 |
| PLpro (6W9C) | 25, 88, -38 | 40, 40, 40 |
| RdRp (6M71) | -18, 28, -39 | 25, 25, 25, |
| Helicase (6JYT) | 410, 35, 67 | 30, 30, 30 |
| nsp10 (6W4H) | 73, 17, 15 | 20, 40, 25 |
| nsp16 (6W4H) | 83, 15, 30 | 25, 30, 20 |
| nsp15 (6VWW) | -90, 22, -38 | 20, 20, 20 |
